# The Development of Diagnostic Reasoning about Uncertain Events between Ages 4–7

**DOI:** 10.1371/journal.pone.0092285

**Published:** 2014-03-20

**Authors:** Christopher D. Erb, David M. Sobel

**Affiliations:** Department of Cognitive, Linguistic, and Psychological Sciences, Brown University, Providence, Rhode Island, United States of America; Durham University, United Kingdom

## Abstract

The present investigation examines the development of children's diagnostic reasoning abilities when such inferences involve belief revision about uncertain potential causes. Four- to 7-year-olds observed an event occur that was due to one of four potential causes. Some of those potential causes were revealed to be efficacious; others were revealed to be inefficacious, but there was always one potential cause presented with unknown efficacy. While all children could make appropriate predictive inferences about this situation, 4- and 5-year-olds were less capable of making correct diagnostic inferences about the cause of the event under these circumstances than older children. We discuss possible mechanisms for this development, as well as speculate on the relation between these findings and literature in children's scientific reasoning.

## Introduction

Diagnostic reasoning involves identifying causes of observed or known effects among a set of possibilities. Such “inference to the best explanation” [1] requires representing potential causes of effects and updating their likelihood based on data. Appropriate diagnosis requires one to appreciate the causal structure of the environment in order to represent the possible candidate causes and weigh them against one another in a rational manner.

How early does diagnostic reasoning emerge in children? Several studies suggest that children can engage in diagnostic reasoning about causal structures at early ages [2]–[5]. This previous research suggests that such capabilities are present during the first year of life [6]–[8]. By the preschool years, children's actions suggest they engage in hypothesis testing of the potential causal structures they diagnose, particularly when faced with data that suggest there are multiple possible causal structures [4]–[5], [9]–[11]. The conclusion one might draw from this evidence is that young children can diagnose causal structures from observed data, and that diagnostic capacities are present quite early in development.

The majority of these studies, however, require children to engage in a specific kind of diagnostic inference – one in which children know, or can infer the efficacy of each possible cause of the outcome. For instance, in one study [5] 4-year-olds were initially presented with ambiguous data, then showed them data that resolved the ambiguity, such that they knew or could infer the efficacy of each individual cause, and demonstrated that children made appropriate diagnostic inferences (younger children could make similar inferences as well, see [12]). Similarly, previous research [4] asked 4- and 5-year-olds to diagnose an uncertain causal structure after they had the opportunity to intervene on the structure in the form of free play. Some children generated the individual efficacy of each potential cause, and these children were likely to diagnose the structure accurately. Other children did not generate these data, and these children were less likely to do so.

What is interesting about these studies is that children are never presented with cases in which the efficacy of at least some potential causes is unknown. Our lab directly examined this issue and found that 3- and 4-year-olds had little difficulty making diagnostic inferences on trials in which children had observed the efficacy of all the potential causes [13]. However, preschoolers struggled with cases in which they did not know whether certain events had efficacy.

For example, children 3 and 4 years of age were introduced to a machine that lit up and played music when certain objects were placed on it (see Experiment 3 of [13]). Children first observed a set of three objects. Each was demonstrated on the machine and some (either 1 or 2 of the 3) activated it. A fourth object was then placed on the table and it was never demonstrated on the machine, so its efficacy was unknown.

The experimenter then occluded the objects and machine from the child, and told the child she was placing an object on the machine. The machine's music was played, giving the illusion that one of the objects was placed on the machine. The music stopped, and the experimenter gave the appearance she was moving an object back to its original location. The occluder was then removed and the experimenter explained to the child that she used one of the four objects to make the machine go. She asked children which object they believed was used to activate the machine. Regardless of their response, children were told they were wrong, and were asked to make another response from the set of three objects remaining on the table.

Children were coded as to whether they made an “error-free” response – that is, whether they chose an object they had previously seen fail to activate the machine during the demonstration in response to either of the test questions. Overall, 3-year-olds responded to these questions no differently from chance. Four-year-olds were not significantly better, although their overall level of performance exceeded chance-level responding (i.e., they generated an error free response on 50% of the trials). Children's poor performance was not the result of their failing to remember the object's efficacy during the demonstration (they responded at near-ceiling levels on such control questions). Moreover, in a subsequent experiment, when the fourth object's efficacy was demonstrated to the child, error-free inferences in both age groups increased.

Even though 4-year-olds responded better than chance expectations, their overall level of performance was not close to ceiling. Indeed, other measures of diagnostic inference that require the child to reason about potential causes with unknown efficacy suggest that such abilities do not develop until much later. For instance, previous research found that 4–5-year-olds struggled on diagnostic problems that involved uncertainty [14]. This paper found that it was not until children were 9–10 that they responded at ceiling levels on these questions.

The experiments in this previous study [14], however, were highly contextualized and presented children with complex verbal problems, which may have underestimated children's reasoning capacities. Indeed, there is some evidence that children understand uncertainty, at least in their probabilistic reasoning, at slightly earlier ages. Some researchers [15] have suggested that preoperational children's choices on various probabilistic tasks were arbitrary, and that it was not until the concrete operational stage that children could differentiate between deterministic and probabilistic relations. Similar findings come from experiments in which 4- to 10-year-olds were presented with spinners that were divided into different areas of black and white [16]. In these experiments, children were shown a spinner and asked to predict the color where the pointer would land. Children under age 6 chose colors randomly, regardless of the configuration of the spinner, whereas older children demonstrated an emerging understanding of probability (see [17] for similar conclusions, and [18] for evidence that 5-year-olds, but not 4-year-olds might have similar capacities). These data suggest that an understanding of uncertainty in inference develops after the preschool years. Our question is whether such findings reflect children's capacity to make diagnostic inferences about uncertain events.

The present experiment uses the method developed in previous work from our lab [13] to examine whether children's capacities for diagnostic inference about uncertain potential causes continues to develop beyond the preschool years, specifically focusing on differences between preschoolers and 6–7-year-olds. Children between the ages of 4–7 were introduced to a machine that lights up and plays music when certain objects are placed on it (a “blicket detector” [19]). They observed a set of three objects. Some activated the machine; some did not. A fourth object of unknown causal efficacy was also present (either presented with the other objects or introduced after the other objects' efficacy had been demonstrated). On some trials, children were then asked to predict whether the machine would activate if those objects were placed on it. On other trials, children were asked to diagnose which object was used to activate the machine.

## Experiment

### Method

#### Participants

The final sample included 95 children from two age groups: 4–5-year-olds (*n* = 48, 32 girls, 16 boys, *M* = 60.26 months, range = 48.20–71.60 months) and 6–7-year-olds (*n* = 47, 23 girls, 24 boys, *M* = 83.79 months, range  = 72.00–98.50 months). Children were recruited through a list of birth records, flyers posted at local preschools, or at a local children's museum. Most children were Caucasian and appeared to come from middle to upper-middle class families; however, no specific indicator of SES was obtained.

#### Materials

The machine was a 20.3 cm×15.2 cm×7.6 cm black plastic box with a pressure sensitive white plate on top. Under the white plate was a set of LCD lights that were visible through the plate when the machine was activated. A button on a remote control was used (out of sight of the child) to activate the machine. Whenever the button was pressed the machine would light up and play music. The machine was battery-powered, so no external cords ran to or from it.

Four sets of four wooden blocks were also used. In each set, the blocks were all the same shape (cubes, cuboids, triangular-based pyramids, or cylinders; all approximately 3–6 cm in height), and each of the sixteen objects was painted a different color. A 56 cm×43 cm piece of cardboard was also used to temporarily occlude view of the machine and blocks.

#### Procedure

The procedure used here was approved by the Institutional Review Board of Brown University. Written permission to conduct this research was obtained from parents/guardians of all child participants in the study. Our procedure was based on that of previous research from our lab (Experiment 3 of [13]). All children were tested in the laboratory or in a quiet room at the museum by a male experimenter. The machine was placed on the table, and the experimenter told the child that they would play a game with the machine and some toys. They were told the machine was special, because some toys made the machine light up and play music and some toys did not.

All children were presented with four different trials, presented in a random order. Two were *prediction* trials, in which children observed the causal efficacy of a subset of the blocks and had to predict what would happen when each block was placed on the machine subsequently. These trials acted as control trials for the two *diagnostic* trials, in which children observed the causal efficacy of a subset of the blocks, and then that the machine activated while the block set and machine were occluded. Children were asked to determine which block had been used to activate the machine. Critically, the prediction trials ensured that children could remember the efficacy of the blocks that were demonstrated to the child.

In each trial, the experimenter introduced the child to four blocks identical in shape but different in color. Next, he demonstrated the efficacy of three of the blocks twice (the *known* blocks), while one block's efficacy was never demonstrated (the *unknown* block). In the *one-cause* predictive and diagnostic trials, only one of the three known blocks activated the machine. In the *two-cause* predictive and diagnostic trials, two of the three known blocks activated the machine. The two-cause trial did not involve combined or interactive effects.

After the objects had been introduced and demonstrated, the experimenter occluded the machine and objects from the child's view and said, “I'm going to put one of these objects on the machine, so listen very carefully.” The experimenter mimed placing one of the objects on the machine behind the occluder and used the remote control, which was held in his hand hidden from the child to “activate” the machine. Thus, the sound the child heard was of an object going on the machine, and the machine activating. The experimenter emphasized this by saying, “Do you hear that? The machine is going, isn't it?” Note that none of the blocks changed position on the table. The experimenter then hid the remote control (miming returning an object to its original location) and removed the occluder, so that the child could see the four blocks and the machine.

On the predictive trials, the experimenter then asked the child whether each of the four blocks would activate the machine if the block were placed on top of it: “If I put this one on the machine, will it make the machine go?” On the diagnostic trials, he asked, “Which one of the toys did I just put on the machine when I had this <indicating the occluder> between us?” After the child indicated a response the experimenter said, “That's a really good guess, but it wasn't this one.” He removed the selected block from the table and asked a belief revision question, “Which one do you think it was?” After the child selected a second block, the experimenter provided non-evaluative feedback and moved onto the next trial.

One difference between the procedure used here and the procedure used in the previous study [13] is that the occluder was used on both types of trials, not just diagnostic trials. We made this modification to ensure that the procedure used in the predictive and diagnostic trials was as similar as possible. A second modification was that we varied how the unknown block was introduced. Approximately half of the children in each age group were shown all four blocks at the same time (the *all-together* condition). The other group of children in each age group was shown the three known blocks first. After the efficacy of these three blocks was each demonstrated to the child, the fourth unknown block was added to the table (the *separate* condition).

Previous work only presented children with a procedure that mimicked the separate condition, so this was a direct replication of that study [13]. Our rationale for manipulating the presentation of the known and unknown blocks was to eliminate a potential confound in the procedure used in the previous study. In that procedure, children might have thought the unknown block was distinctly separate from the known blocks because it was introduced later. As a result, children may have preferentially selected or avoided the unknown block when making a diagnosis.

Children's participation in the study was videotaped and scored by undergraduate research assistants, blind to the hypotheses of the experiments. The first author supervised this data analysis, and then independently performed a data analysis himself to ensure reliability. As per the IRB of the authors' university, video data will be stored for 5 years following publication of this article. As per the IRB of the authors' home university, parents have the option to make their video data available to other researchers. Such data is available on request, assuming legitimate research reasons, and IRB approval from the authors' home university.

## Results

Table 1 shows the percentage of trials on which children did not make an error in responding to each of the predictive or diagnostic questions. On the prediction trials, an error included stating that a known block that activated the machine would no longer do so or that a known block that did not activate the machine would do so. Responses to the unknown block on the prediction trials were not included in the subsequent analyses. Given that there was no normatively correct response on these trials, responses of “Yes”, “No”, and “I don't know” were accepted (i.e., the experimenter continued on to the next question), but these responses were not scored. On the diagnostic trials, an error indicated that children chose a block that was demonstrated to not have efficacy in response to either of the test questions.

**Table 1:** Proportion of Error-Free Trials across the All-Together and Separate Conditions.

	Predictive				Diagnostic			
	One-Cause		Two-Cause		One-Cause		Two-Cause	
Age in Years	4–5	5–6	4–5	5–6	4–5	5–6	4–5	5–6
All-Together	0.96 (0.20)	0.83 (0.38)	0.88 (0.34)	1.00 (0.00)	0.75 (0.44)	0.88 (0.34)	0.92 (0.28)	0.92 (0.28)
Separate	0.88 (0.38)	0.91 (0.29)	0.75 (0.44)	0.91 (0.29)	0.63 (0.49)	0.91 (0.29)	0.83 (0.39)	0.91 (0.29)
Combined	0.92 (0.28)	0.87 (0.34)	0.81 (0.39)	0.96 (0.20)	0.69 (0.47)	0.89 (0.31)	0.88 (0.33)	0.91 (0.28)

*Notes*. Standard deviations shown in parentheses. All-together (*N* = 48), Separate (*N* = 47).

We first considered responses to the prediction trials. There was no difference in the number of children who erred on the one-cause and the two-cause trials, χ^2^(1, *N* = 95)  = 0.00, *p* = 1.00, so we combined these data in the subsequent analyses. There was no difference between the separate and all-together conditions, χ^2^(1, *N* = 95)  = 1.12, *p* = 0.29, nor was there a difference between the two age groups, χ^2^(1, *N* = 95)  = 0.91, *p* = 0.34. Most children (75 of the 95 children or 79% of the sample) responded without error on both of these trials. Overall, responses were well above chance levels (25%), Binomial test, *p*<0.01. These data indicate that children remembered the efficacy of the known objects. For the unknown object, children said it had efficacy 49% of the time across the two trials, that it did not have efficacy 25% of the time, and that they did not know 26% of the time. This was not an even distribution, Friedman χ^2^(2, *N* = 95)  = 20.23, *p*<0.01, but there was no difference in how children responded between the two age groups to these questions, all χ^2^(1, *N* = 95)-values <1.30, all *p*-values >0.25.

We next considered whether children made accurate responses to the diagnostic trials. Here, there was a difference in performance between the one-cause and two-cause trials, McNemar χ^2^(1, *N* = 95)  = 4.05, *p* = 0.04, so we analyzed these trials separately. There was no difference between the separate and all-together conditions on the one-cause or the two-cause trials, both Fisher Exact tests, *p*-values >0.51. Thus, we combined the data from these two conditions.

Responses on the two-cause trial did not differ between the younger (88% error-free responses) and older (91%) children, Fisher Exact test, *p* = 0.74. Responses on the one cause trial did differ, with the younger children less accurate (69%) than the older children (89%), Fisher's Exact test (one-tailed), *p* = 0.015. Both age groups, however, were accurate above chance levels on both trials (16% for the one-cause trials and 50% for the two-cause trials), Binomial tests, all *p*-values <0.01.

We were also interested in whether there was a relation between the prediction and diagnostic trials. When we considered only the 75 children who responded without error on the two prediction trials, the basic findings reported above still held: There was no difference between the age groups on the two-cause diagnostic trials. Younger children were less accurate on the one-cause trial than the older children (72% vs. 90%), Fisher's Exact test (one-tailed), *p* = 0.049. Both age groups were significantly above chance on both trials, Binomial tests, all *p*-values <0.01. We also examined how responses to the unknown object on the prediction trials related to accurate responses on the diagnostic trials. We found no significant relation on the one cause trial. On the two-cause trial, there was one trend: the more children stated the unknown block would activate the machine, the less likely they were respond accurately on this trial, ρ(93)  = -0.185, *p* = 0.075. We suspect this is a random occurrence.

Finally, we considered how children specifically responded on the diagnostic trials, both when they were accurate and when they were inaccurate. When children erred on the diagnostic trials, how did they do so? We first considered whether diagnostic errors were made in response to the first or the second test question. We found that the majority of errors occurred in response to the second test question, for both the one-cause (80% of the time) and two-cause trials (70% of the time). This is somewhat different from the results of the previous study [13], which found a similar result for the one-cause trials (78% of the time), but that the majority of errors on the two-cause trials were on the *first* test question (69% of the time). The authors argued that in this condition, children simply could not track which objects were known and efficacious vs. unknown (and thus possibly efficacious), and just responded randomly. This pattern of responses might have been more descriptive of the younger children (i.e., 3-year-olds) that they tested, but we do not believe this interpretation reflects older children's capacities.

Perhaps more interesting is how children responded when they were accurate. On the one-cause trial, this indicated that they chose the object that they had seen activate the machine and the unknown object in response to the two test questions. The order in which they did this, however, was not evenly distributed. Of the 75 children who responded accurately to this trial, 52 of them (69%) chose the known object first and the unknown object second, Binomial test, *p*<0.01. Choosing the unknown object first when making a correct response showed a trend to be more likely with age, ρ(73)  = 0.20, *p* = 0.07.

On the two-cause trial, there were six possible ways to respond accurately: choosing the two objects with known efficacy (in either order), choosing either of the objects with known efficacy, and then the unknown one, or choosing the unknown object, and then either of the known objects. Again, these response types were not evenly distributed, with children choosing the two known objects 59% of the time among the children who responded accurately on this trial. This was more often than the other four possibilities, Binomial test, *p*<0.01. That is, in response to this trial, children often did not choose the unknown block. However, there was no relation between whether children did so and their age, ρ(83)  = 0.06, *p* = 0.58.

## Discussion

The present experiment showed that children's diagnostic reasoning about uncertain potential causes undergoes development between the ages of 4–7. In particular, 6- and 7-year-olds were better than younger children at making a particular kind of diagnostic inference – one that specifically required recognizing that an object whose causal efficacy was unknown could be efficacious. We found that older children were less likely to err on the one-cause diagnostic trials, which specifically required children to choose the unknown block as a potential cause. The younger children did respond above chance on these trials, as did 4-year-olds on similar trials in previous work [13]. The significant contribution of the present experiment is that preschoolers' diagnostic capacities are not fully developed. Only by the time children are 6, they have acquired the capacity to represent that an object whose efficacy has not been demonstrated could be a potential cause.

But what exactly is developing? One possibility is that children's capacity to remember the blocks' efficacy develops. This would allow older children to make more accurate responses on the diagnostic trials. This possibility, however, seems unlikely. All children, including the younger ones, were well above chance on the prediction trials, suggesting that even the youngest children could recall which objects had made the machine activate. We also observed the same development difference on the diagnostic trials when we only considered children who responded without error on the prediction trials. Further, one could argue that the all-together condition placed fewer memory demands on the child than the separate condition, as the child had to track only the efficacy of the blocks (while in the separate condition, they had to track efficacy as well as which block was introduced after that efficacy was demonstrated). Because there was no difference in performance between the two conditions, children's memory capacities seem not to be mediating the development we observed.

Another possibility is that children made a specific inference about the efficacy of the unknown block in the diagnostic trials (which is reflected in their responses about the unknown object in the prediction trials). That is, children explicitly reasoned as if that object did or did not have efficacy. This could have led children to make more errors in the one-cause trials, specifically if they explicitly believed that it did not have efficacy. Again, we think this possibility is unlikely. We examined how children responded to the unknown object on the prediction trials, and there was no difference between the older and the younger children. We also examined whether these judgments related to error-free performance on the one-cause diagnostic trials, where we observed the developmental difference; they did not.

Rather, we argue that children are developing a specific inferential capacity between the ages of 4 and 6. By age 6, they appreciate than a potential cause whose efficacy is unknown *could* be efficacious (that is, it might be or it might not be). Such development is consistent with the literature on probabilistic reasoning presented in the introduction (e.g., [16]). But more relevantly, such development is analogous to inferences observed in the literature on children's counterfactual reasoning. For instance, by the age of 4 children can reason about a specific counterfactual involving two outcomes (given that outcome A occurred between possible outcomes, children reason that if outcome A had not occurred, outcome B did) [20]. Only at age 6, however, can children recognize that it was necessary to prepare for both outcomes A and B given that the cause occurred [20]. That is, only by age 6 do children prepare for the outcomes of events that might have been.

We believe the present findings are highly consistent with these results; inferences about uncertainty are not just predictive in nature, but relate to children's capacity for diagnosis as well. Thus, young children, perhaps even infants, have causal reasoning abilities, including the capacity to engage in diagnostic inference. However, the kinds of diagnostic inferences they can make are limited to cases in which all of the possible effects are known or can be inferred from evidence. Where young children struggle is with diagnostic inferences that require representing that events whose efficacy they have never observed could be causes of an observed effect.

Support for this hypothesis comes from considering how children responded accurately to both diagnostic inferences. On the one-cause trial, where children had to choose the unknown block to be accurate, more children chose it in response to the second question than the first. Similarly, on the two-cause trial, children who responded correctly often avoided choosing the unknown object. These data suggest that children initially used their observations to respond to these questions, and only considered the possibility that the unknown object could be a cause if necessary. Critically, the trend that older children treated the unknown object equivalently to the known object in the one cause condition indicates that they might have believed that it was equally likely to be a potential cause.

There is one other possibility, which is that children were capable of recognizing that the unknown object could have activated the machine, but they were unable to change their initial response, and defaulted to guessing. That is, once children made a response to the first test question, they could not revise their response once they were told that they were incorrect. As such, they defaulted to responding randomly on the second test question. Indeed, when accuracy was examined for only the first test question, children of all ages were ∼96% correct, and when children erred, most of those errors were on the second test question. However, on the one-cause diagnostic trials, when children responded to the first test question without making an error, they made an error-free response on 82% of the second test questions, significantly greater than chance responding (33%, Binomial test, *p*<.01). There was a developmental difference here, with 4–5-year-olds making error free responses 73% of the time and 6–7-year-olds making error free responses 91% of the time. This difference was significant, Fisher's Exact Test (one-tailed), *p* = 0.023. This suggests that children's difficulty was not specifically with changing their response in the face of negative evidence.

We conclude by suggesting that the present experiment has bearing not only on the literature on children's causal inferences, but on their developing scientific reasoning as well. There are many differences between the blicket detector paradigm used here and previous investigations of scientific inference, and we do not wish to discount those differences. However, we do want to focus on one important similarity: Explicit scientific reasoning often requires the type of diagnostic inference we are investigating (albeit presented in quite a different context). For instance, experiments testing the ‘Control of Variables’ strategy (e.g., [21]) involve determining the efficacy of individual causes of an observed event, given that the individual efficacy of the causes was originally unknown, and that some causes were potentially manipulated or held constant. This strategy is particularly difficult for young children in situations where they can manipulate potential causes. Indeed, many scientific reasoning studies suggest that such diagnostic inference is difficult not only for preschoolers, but even older children as well [22]–[28].

One way of bridging the gap between these two bodies of research is to consider the exact nature of the inference children have to make. Much of the research suggesting that young children have sophisticated causal reasoning abilities involves relatively simple cases of diagnostic inference, cases in which the child knows the efficacy of (usually only two) potential causes. Moreover, these investigations rarely require the child to explicitly make more than one inference (i.e., children are almost never told they are incorrect, and must infer a different conclusion from the same data, as we did here). In contrast, experiments on children's scientific reasoning often present participants with multiple potential causes whose individual efficacy is unknown, and require them to update existing beliefs about a causal structure given novel evidence that those beliefs are wrong. There is no question that scientific reasoning involves many other cognitive capacities that are developing during (and potentially after) the age range we investigated here. But the ability to engage in this type of diagnostic inference is foundational for such reasoning, and this capacity is developing into the early elementary-school years. We suggest based on this speculation that an important open question for future research is how the emergence of such diagnostic capacities relates to children's explicit ability to engage in scientific reasoning.
